# New records of *Sylvicola* (Diptera: Anisopodidae) from Romania

**DOI:** 10.3897/BDJ.4.e7861

**Published:** 2016-02-08

**Authors:** Levente-Péter Kolcsár, Libor Dvořák, Paul LT Beuk

**Affiliations:** ‡ Babes-Bolyai University, Cluj-Napoca, Romania; § Municipal Museum Mariánské Lázně, Mariánské Lázně, Czech Republic; | Natuurhistorisch Museum Maastricht / Diptera.info, Maastricht, Netherlands

**Keywords:** identification key, window gnats, wood gnats

## Abstract

**Background:**

Anisopodidae (window gnats or wood gnats) is a small family of nematocerous Diptera. Until now only Sylvicola (Anisopus) punctatus (Fabricius, 1787) and Sylvicola (Sylvicola) fenestralis (Scopoli, 1763)​ were reported from Romania.

**New information:**

New faunistic records of *Sylvicola* (Diptera: Anisopodidae) are presented. Sylvicola (Sylvicola) cinctus (Fabricius, 1787) and S. (Anisopus) fuscatus (Fabricius, 1775) are recorded from Romania for the first time. An identification key and illustrations of Romanian *Sylvicola* species are presented.

## Introduction

Anisopodidae (window gnats or wood gnats) is a small family of nematocerous Diptera. Only the genus *Sylvicola* comprising ten species is known from Europe (de Jong 2014). Adults are often collected in forests, but several species are also frequent in semi-opened habitats like bushes, gardens and orchards (Dvořák and Oboňa 2014). The larvae feed in various decaying organic materials, while the adults feed on nectar or similar artificial liquids, like syrup, beer or even meat (Kovář and Barták 2001). These liquids can be used in traps for effective collecting of *Sylvicola* species (Dvořák 2014a, Dvořák 2014b, Kurina 2006, Dvořák and Oboňa 2014).

Until now only two *Sylvicola* species were reported from Romania (Pârvu 2003, Pârvu 2004, Thalhammer 1900). In this study we present an additional two species for the Romanian fauna. An identification key to the Romanian *Sylvicola* species, illustrations of male and female genitalia, phenology and distribution maps of each species also are presented.

## Materials and methods

The Romanian material was collected using different methods. The material deposited in Municipal Museum Mariánské Lázně, Czech Republic (MML) was collected by Gavril Marius Berchi using a beer trap in Caraș-Severin during the period 02-26.06.2014. Material collected by Alexandru Pintilioaie deposited in Maastricht Natural History Museum, Netherlands (NHMM). The remaining material was mainly collected by the first author using an insect net, a Malaise trap or an elderberry (*Sambucus nigra*) syrup trap and finaly stored in 70% ethanol and deposited in the Diptera Collection of the Faculty of Biology and Geology, Cluj-Napoca, Romania (DCBBU). The remainder was collected using different methods (banana trap, hand collecting) by third parties.

The material was identified and the identification key created based on the papers of Haarto (2011), Haenni (1997), Krivosheina and Menzel (1998) and Söli and Rindal (2014).

## Taxon treatments

### Sylvicola (Anisopus) punctatus

(Fabricius, 1787)

#### Materials

- **Type status:** Other material. **Occurrence:** recordedBy: Pintilioaie Alexandru; individualCount: 1; sex: female; **Taxon:** genus: Sylvicola; subgenus: Anisopus; specificEpithet: punctatus; scientificNameAuthorship: (Fabricius, 1787); **Location:** country: Romania; stateProvince: Bacău; municipality: Comăneşti; verbatimElevation: 407 m; verbatimCoordinateSystem: decimal degrees; verbatimSRS: WGS84; decimalLatitude: 46.426079; decimalLongitude: 26.443042; **Event:** eventDate: 28/6/2010; habitat: house garden; **Record Level:** institutionCode: NHMM
- **Type status:** Other material. **Occurrence:** recordedBy: Gavril Marius Berchi; individualCount: 29; sex: females; **Taxon:** genus: Sylvicola; subgenus: Anisopus; specificEpithet: punctatus; scientificNameAuthorship: (Fabricius, 1787); **Location:** country: Romania; stateProvince: Caraș-Severin; municipality: Semenic; locality: Poiana Goznei; verbatimElevation: 1382 m; verbatimCoordinateSystem: decimal degrees; verbatimSRS: WGS84; decimalLatitude: 45.192222; decimalLongitude: 22.071667; **Event:** samplingProtocol: beer trap; eventDate: 02-25/06/2014; habitat: meadow, edge with beech forest; **Record Level:** institutionCode: MML
- **Type status:** Other material. **Occurrence:** recordedBy: Gavril Marius Berchi; individualCount: 1; sex: female; **Taxon:** genus: Sylvicola; subgenus: Anisopus; specificEpithet: punctatus; scientificNameAuthorship: (Fabricius, 1787); **Location:** country: Romania; stateProvince: Caraș-Severin; municipality: Văliug; locality: Valea Râului Bârzava; verbatimElevation: 681 m; verbatimCoordinateSystem: decimal degrees; verbatimSRS: WGS84; decimalLatitude: 45.178056; decimalLongitude: 22.003333; **Event:** samplingProtocol: beer trap; eventDate: 02-25/06/2014; habitat: marsh, edge with beech forest; **Record Level:** institutionCode: MML
- **Type status:** Other material. **Occurrence:** recordedBy: Levente-Péter Kolcsár; individualCount: 1; sex: male; **Taxon:** genus: Sylvicola; subgenus: Anisopus; specificEpithet: punctatus; scientificNameAuthorship: (Fabricius, 1787); **Location:** country: Romania; stateProvince: Cluj; municipality: Cluj-Napoca; locality: Alexandru Borza Botanical Garden; verbatimElevation: 395 m; verbatimCoordinateSystem: decimal degrees; verbatimSRS: WGS84; decimalLatitude: 46.761322; decimalLongitude: 23.586521; **Event:** samplingProtocol: elderberry syrup trap; eventDate: 18-25.06.2015; habitat: garden; **Record Level:** institutionCode: DCBBU

#### Distribution

**Figure 1.**
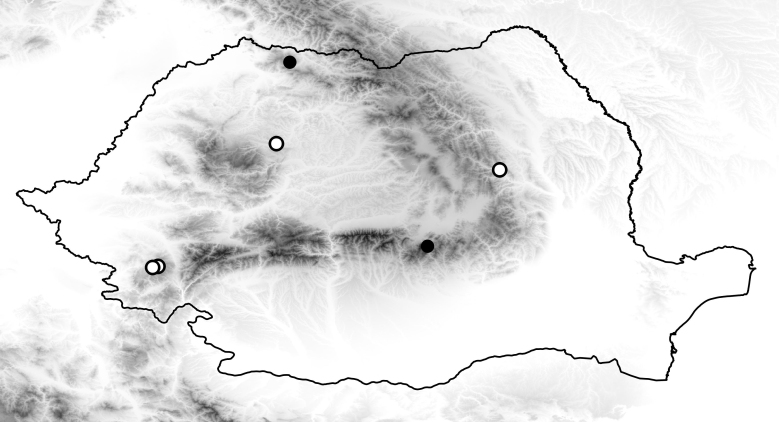
Collecting sites of *Sylvicola punctatus* in Romania. White dots represent new data, black dots are data from literature.

A very common species, widespread in Europe. Known distribution of species in the Romania illustrated in Fig. 1.

#### Notes

Pârvu (2003), Pârvu (2004) listed it incorrectly as *Sylvicola punctata* (Fabricius, 1787).

### Sylvicola (Anisopus) fuscatus

(Fabricius, 1775)

#### Materials

- **Type status:** Other material. **Occurrence:** recordedBy: Levente-Péter Kolcsár; individualCount: 1; sex: male; **Taxon:** genus: Sylvicola; subgenus: Anisopus; specificEpithet: fuscatus; scientificNameAuthorship: (Fabricius, 1775); **Location:** country: Romania; stateProvince: Harghita; municipality: Brădești; locality: Târnava Mare River; verbatimElevation: 487 m; verbatimCoordinateSystem: decimal degrees; verbatimSRS: WGS84; decimalLatitude: 46.331427; decimalLongitude: 25.331074; **Event:** samplingProtocol: insect net; eventDate: 01.05.2010; habitat: willow (*Salix*) forest along river; **Record Level:** institutionCode: DCBBU
- **Type status:** Other material. **Occurrence:** recordedBy: Gavril Marius Berchi; individualCount: 1; sex: female; **Taxon:** genus: Sylvicola; subgenus: Anisopus; specificEpithet: fuscatus; scientificNameAuthorship: (Fabricius, 1775); **Location:** country: Romania; stateProvince: Caraș-Severin; municipality: Văliug; locality: Valea Râului Bârzava; verbatimElevation: 681 m; verbatimCoordinateSystem: decimal degrees; verbatimSRS: WGS84; decimalLatitude: 45.178056; decimalLongitude: 22.003333; **Event:** samplingProtocol: beer trap; eventDate: 02-25/06/2014; habitat: marsh, edge with beech forest; **Record Level:** institutionCode: MML
- **Type status:** Other material. **Occurrence:** recordedBy: Levente-Péter Kolcsár; individualCount: 7; sex: females; **Taxon:** genus: Sylvicola; subgenus: Anisopus; specificEpithet: fuscatus; scientificNameAuthorship: (Fabricius, 1775); **Location:** country: Romania; stateProvince: Cluj; municipality: Cluj-Napoca; locality: Mikó Garden; verbatimElevation: 370 m; verbatimCoordinateSystem: decimal degrees; verbatimSRS: WGS84; decimalLatitude: 46.763666; decimalLongitude: 23.58018; **Event:** samplingProtocol: elderberry syrup trap; eventDate: 18-25.06.2015; habitat: garden; **Record Level:** institutionCode: DCBBU
- **Type status:** Other material. **Occurrence:** recordedBy: Levente-Péter Kolcsár; individualCount: 1; sex: female; **Taxon:** genus: Sylvicola; subgenus: Anisopus; specificEpithet: fuscatus; scientificNameAuthorship: (Fabricius, 1775); **Location:** country: Romania; stateProvince: Cluj; municipality: Cluj-Napoca; locality: Mikó Garden; verbatimElevation: 370 m; verbatimCoordinateSystem: decimal degrees; verbatimSRS: WGS84; decimalLatitude: 46.763666; decimalLongitude: 23.58018; **Event:** samplingProtocol: elderberry syrup trap; eventDate: 01-03.06.2015; habitat: garden; **Record Level:** institutionCode: DCBBU
- **Type status:** Other material. **Occurrence:** recordedBy: Levente-Péter Kolcsár; individualCount: 1; sex: female; **Taxon:** genus: Sylvicola; subgenus: Anisopus; specificEpithet: fuscatus; scientificNameAuthorship: (Fabricius, 1775); **Location:** country: Romania; stateProvince: Cluj; municipality: Cluj-Napoca; locality: Mikó Garden; verbatimElevation: 370 m; verbatimCoordinateSystem: decimal degrees; verbatimSRS: WGS84; decimalLatitude: 46.763666; decimalLongitude: 23.58018; **Event:** samplingProtocol: insect net; eventDate: 16-23.06.2015; habitat: garden; **Record Level:** institutionCode: DCBBU
- **Type status:** Other material. **Occurrence:** recordedBy: Levente-Péter Kolcsár; individualCount: 11; sex: 2 males, 9 females; **Taxon:** genus: Sylvicola; subgenus: Anisopus; specificEpithet: fuscatus; scientificNameAuthorship: (Fabricius, 1775); **Location:** country: Romania; stateProvince: Cluj; municipality: Cluj-Napoca; locality: Alexandru Borza Botanical Garden; verbatimElevation: 395 m; verbatimCoordinateSystem: decimal degrees; verbatimSRS: WGS84; decimalLatitude: 46.761322; decimalLongitude: 23.586521; **Event:** samplingProtocol: elderberry syrup trap; eventDate: 18-25.06.2015; habitat: garden; **Record Level:** institutionCode: DCBBU
- **Type status:** Other material. **Occurrence:** recordedBy: Edina Török; individualCount: 1; sex: female; **Taxon:** genus: Sylvicola; subgenus: Anisopus; specificEpithet: fuscatus; scientificNameAuthorship: (Fabricius, 1775); **Location:** country: Romania; stateProvince: Cluj; municipality: Cluj-Napoca; locality: Mikó Garden; verbatimElevation: 370 m; verbatimCoordinateSystem: decimal degrees; verbatimSRS: WGS84; decimalLatitude: 46.763666; decimalLongitude: 23.58018; **Event:** samplingProtocol: insect net; eventDate: 16.05.2015; habitat: garden; **Record Level:** institutionCode: DCBBU

#### Distribution

**Figure 2.**
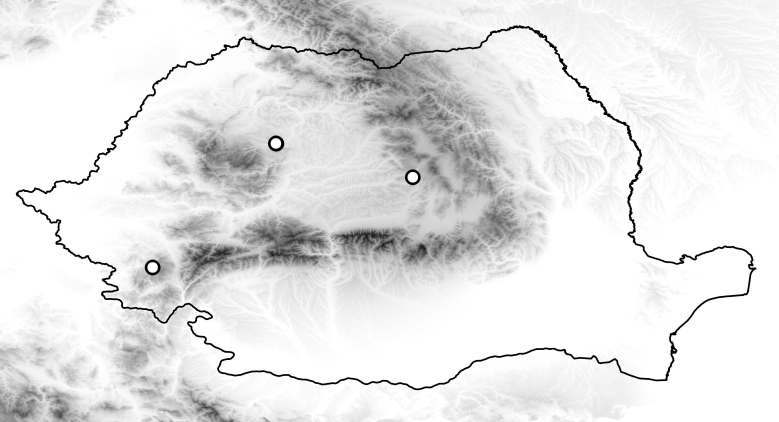
Collecting sites of *Sylvicola fuscatus* in Romania.

Known distribution of species in the Romania illustrated i n Fig. 2.

#### Notes

First records from Romania.

### Sylvicola (Sylvicola) cinctus

(Fabricius, 1787)

#### Materials

- **Type status:** Other material. **Occurrence:** recordedBy: Pintilioaie Alexandru; individualCount: 3; sex: 1 male, 2 females; **Taxon:** genus: Sylvicola; subgenus: Sylvicola; specificEpithet: cinctus; scientificNameAuthorship: (Fabricius, 1787); **Location:** country: Romania; stateProvince: Bacău; municipality: Comăneşti; verbatimElevation: 413 m; verbatimCoordinateSystem: decimal degrees; verbatimSRS: WGS84; decimalLatitude: 46.426082; decimalLongitude: 26.442495; **Event:** samplingProtocol: Banana trap; eventDate: 28.03.2010; habitat: house garden; **Record Level:** institutionCode: NHMM
- **Type status:** Other material. **Occurrence:** recordedBy: Pintilioaie Alexandru; individualCount: 5; sex: 3 males, 2 females; **Taxon:** genus: Sylvicola; subgenus: Sylvicola; specificEpithet: cinctus; scientificNameAuthorship: (Fabricius, 1787); **Location:** country: Romania; stateProvince: Bacău; municipality: Comăneşti; verbatimElevation: 413 m; verbatimCoordinateSystem: decimal degrees; verbatimSRS: WGS84; decimalLatitude: 46.426082; decimalLongitude: 26.442495; **Event:** samplingProtocol: Banana trap; eventDate: 31.03.2010; habitat: house garden; **Record Level:** institutionCode: NHMM
- **Type status:** Other material. **Occurrence:** recordedBy: Levente-Péter Kolcsár; individualCount: 1; sex: male; **Taxon:** genus: Sylvicola; subgenus: Sylvicola; specificEpithet: cinctus; scientificNameAuthorship: (Fabricius, 1787); **Location:** country: Romania; stateProvince: Harghita; municipality: Brădești; locality: Târnava Mare River; verbatimElevation: 487 m; verbatimCoordinateSystem: decimal degrees; verbatimSRS: WGS84; decimalLatitude: 46.331427; decimalLongitude: 25.331074; **Event:** samplingProtocol: insect net; eventDate: 01.05.2010; habitat: willow (*Salix*) forest along river; **Record Level:** institutionCode: DCBBU
- **Type status:** Other material. **Occurrence:** recordedBy: Pintilioaie Alexandru; individualCount: 3; sex: 2 males, 1 females; **Taxon:** genus: Sylvicola; subgenus: Sylvicola; specificEpithet: cinctus; scientificNameAuthorship: (Fabricius, 1787); **Location:** country: Romania; stateProvince: Bacău; municipality: Comăneşti; verbatimElevation: 413 m; verbatimCoordinateSystem: decimal degrees; verbatimSRS: WGS84; decimalLatitude: 46.426082; decimalLongitude: 26.442495; **Event:** samplingProtocol: at light; eventDate: 20.10.2010; habitat: house garden; **Record Level:** institutionCode: NHMM
- **Type status:** Other material. **Occurrence:** recordedBy: Gavril Marius Berchi; individualCount: 1; sex: female; **Taxon:** genus: Sylvicola; subgenus: Sylvicola; specificEpithet: cinctus; scientificNameAuthorship: (Fabricius, 1787); **Location:** country: Romania; stateProvince: Caraș-Severin; municipality: Semenic; locality: Poiana Muntelui; verbatimElevation: 1381 m; verbatimCoordinateSystem: decimal degrees; verbatimSRS: WGS84; decimalLatitude: 45.17500; decimalLongitude: 22.062778; **Event:** samplingProtocol: beer trap; eventDate: 02-25/06/2014; habitat: peat bog; **Record Level:** institutionCode: MML
- **Type status:** Other material. **Occurrence:** recordedBy: Gavril Marius Berchi; individualCount: 29; sex: females; **Taxon:** genus: Sylvicola; subgenus: Sylvicola; specificEpithet: cinctus; scientificNameAuthorship: (Fabricius, 1787); **Location:** country: Romania; stateProvince: Caraș-Severin; municipality: Semenic; locality: Poiana Goznei; verbatimElevation: 1382 m; verbatimCoordinateSystem: decimal degrees; verbatimSRS: WGS84; decimalLatitude: 45.192222; decimalLongitude: 22.071667; **Event:** samplingProtocol: beer trap; eventDate: 02-25/06/2014; habitat: meadow, edge with beech forest; **Record Level:** institutionCode: MML
- **Type status:** Other material. **Occurrence:** recordedBy: Gavril Marius Berchi; individualCount: 1; sex: female; **Taxon:** genus: Sylvicola; subgenus: Sylvicola; specificEpithet: cinctus; scientificNameAuthorship: (Fabricius, 1787); **Location:** country: Romania; stateProvince: Caraș-Severin; municipality: Văliug; locality: Valea Râului Bârzava; verbatimElevation: 681 m; verbatimCoordinateSystem: decimal degrees; verbatimSRS: WGS84; decimalLatitude: 45.178056; decimalLongitude: 22.003333; **Event:** samplingProtocol: beer trap; eventDate: 02-25/06/2014; habitat: marsh, edge with beech forest; **Record Level:** institutionCode: MML
- **Type status:** Other material. **Occurrence:** recordedBy: Levente-Péter Kolcsár; individualCount: 2; sex: females; **Taxon:** genus: Sylvicola; subgenus: Sylvicola; specificEpithet: cinctus; scientificNameAuthorship: (Fabricius, 1787); **Location:** country: Romania; stateProvince: Cluj; municipality: Cluj-Napoca; locality: Alexandru Borza Botanical Garden; verbatimElevation: 395 m; verbatimCoordinateSystem: decimal degrees; verbatimSRS: WGS84; decimalLatitude: 46.761322; decimalLongitude: 23.586521; **Event:** samplingProtocol: Malaise trap; eventDate: 18-25.06.2015; habitat: garden; **Record Level:** institutionCode: DCBBU
- **Type status:** Other material. **Occurrence:** recordedBy: Levente-Péter Kolcsár; individualCount: 1; sex: female; **Taxon:** genus: Sylvicola; subgenus: Sylvicola; specificEpithet: cinctus; scientificNameAuthorship: (Fabricius, 1787); **Location:** country: Romania; stateProvince: Harghita; municipality: Sânmartin; locality: Rugat Valley; verbatimElevation: 950 m; verbatimCoordinateSystem: decimal degrees; verbatimSRS: WGS84; decimalLatitude: 46.26702; decimalLongitude: 26.010467; **Event:** samplingProtocol: insect net; eventDate: 25.08.2014; habitat: spruce (*Picea*) forest; **Record Level:** institutionCode: DCBBU
- **Type status:** Other material. **Occurrence:** recordedBy: Edina Török; individualCount: 1; sex: male; **Taxon:** genus: Sylvicola; subgenus: Sylvicola; specificEpithet: cinctus; scientificNameAuthorship: (Fabricius, 1787); **Location:** country: Romania; stateProvince: Sălaj; municipality: Gâlgău Almașului; locality: Grădina Zmeilor; verbatimElevation: 290 m; verbatimCoordinateSystem: decimal degrees; verbatimSRS: WGS84; decimalLatitude: 47.199465; decimalLongitude: 23.304763; **Event:** samplingProtocol: insect net; eventDate: 21.02.2015; habitat: oak (Quercus) forest; **Record Level:** institutionCode: DCBBU
- **Type status:** Other material. **Occurrence:** recordedBy: Levente-Péter Kolcsár; individualCount: 2; sex: females; **Taxon:** genus: Sylvicola; subgenus: Sylvicola; specificEpithet: cinctus; scientificNameAuthorship: (Fabricius, 1787); **Location:** country: Romania; stateProvince: Caraș-Severin; municipality: Poiana Mărului; locality: Valea Bistra Mărului; verbatimElevation: 1444 m; verbatimCoordinateSystem: decimal degrees; verbatimSRS: WGS84; decimalLatitude: 45.348317; decimalLongitude: 22.646293; **Event:** samplingProtocol: insect net; eventDate: 02.05.2015; habitat: spruce (*Picea*) forest; **Record Level:** institutionCode: DCBBU
- **Type status:** Other material. **Occurrence:** recordedBy: Levente-Péter Kolcsár; individualCount: 1; sex: female; **Taxon:** genus: Sylvicola; subgenus: Sylvicola; specificEpithet: cinctus; scientificNameAuthorship: (Fabricius, 1787); **Location:** country: Romania; stateProvince: Cluj; municipality: Cluj-Napoca; locality: Mikó Garden; verbatimElevation: 370 m; verbatimCoordinateSystem: decimal degrees; verbatimSRS: WGS84; decimalLatitude: 46.763666; decimalLongitude: 23.58018; **Event:** samplingProtocol: insect net; eventDate: 16.05.2015; habitat: garden; **Record Level:** institutionCode: DCBBU
- **Type status:** Other material. **Occurrence:** recordedBy: Levente-Péter Kolcsár; individualCount: 1; sex: female; **Taxon:** genus: Sylvicola; subgenus: Sylvicola; specificEpithet: cinctus; scientificNameAuthorship: (Fabricius, 1787); **Location:** country: Romania; stateProvince: Cluj; municipality: Cluj-Napoca; locality: Mikó Garden; verbatimElevation: 370 m; verbatimCoordinateSystem: decimal degrees; verbatimSRS: WGS84; decimalLatitude: 46.763666; decimalLongitude: 23.58018; **Event:** samplingProtocol: insect net; eventDate: 26.04.2015; habitat: garden; **Record Level:** institutionCode: DCBBU
- **Type status:** Other material. **Occurrence:** recordedBy: Levente-Péter Kolcsár; individualCount: 1; sex: female; **Taxon:** genus: Sylvicola; subgenus: Sylvicola; specificEpithet: cinctus; scientificNameAuthorship: (Fabricius, 1787); **Location:** country: Romania; stateProvince: Brașov; municipality: Sâmbăta de Sus; locality: Sâmbăta Valley; verbatimElevation: 1092 m; verbatimCoordinateSystem: decimal degrees; verbatimSRS: WGS84; decimalLatitude: 45.646639; decimalLongitude: 24.791212; **Event:** samplingProtocol: insect net; eventDate: 29.05.2014; habitat: spruce (*Picea*) forest; **Record Level:** institutionCode: DCBBU
- **Type status:** Other material. **Occurrence:** recordedBy: Levente-Péter Kolcsár; individualCount: 1; sex: female; **Taxon:** genus: Sylvicola; subgenus: Sylvicola; specificEpithet: cinctus; scientificNameAuthorship: (Fabricius, 1787); **Location:** country: Romania; stateProvince: Cluj; municipality: Cluj-Napoca; locality: Alexandru Borza Botanical Garden; verbatimElevation: 395 m; verbatimCoordinateSystem: decimal degrees; verbatimSRS: WGS84; decimalLatitude: 46.761322; decimalLongitude: 23.586521; **Event:** samplingProtocol: Malaise trap; eventDate: 11.12.2015; habitat: garden; **Record Level:** institutionCode: DCBBU
- **Type status:** Other material. **Occurrence:** recordedBy: Levente-Péter Kolcsár; individualCount: 1; sex: male; **Taxon:** genus: Sylvicola; subgenus: Sylvicola; specificEpithet: cinctus; scientificNameAuthorship: (Fabricius, 1787); **Location:** country: Romania; stateProvince: Brasov; municipality: Zarnesti; locality: Plaiul Foii hut; verbatimElevation: 875 m; verbatimCoordinateSystem: decimal degrees; verbatimSRS: WGS84; decimalLatitude: 45.560103; decimalLongitude: 25.197629; **Event:** samplingProtocol: Malaise trap; eventDate: 18-25.06.2015; habitat: garden; **Record Level:** institutionCode: DCBBU

#### Distribution

**Figure 3.**
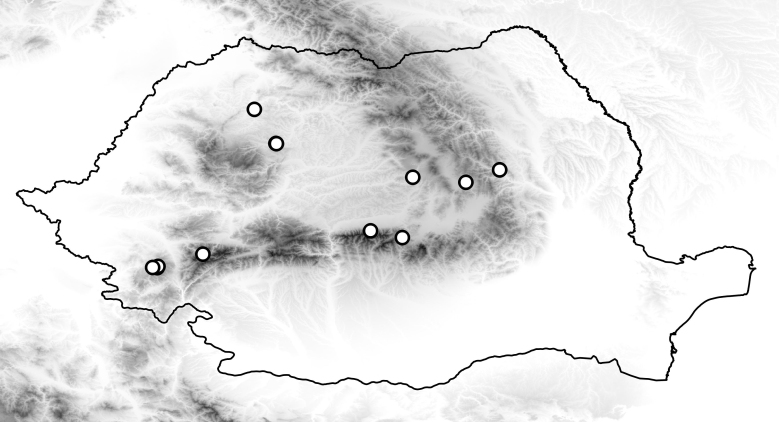
Collecting sites of *Sylvicola cinctus* in Romania.

Common species, known from almost Europe. Known distribution of the species in the Romania illustrated on Fig. 3.

#### Notes

The record of *S. fenestralis* (Scopoli, 1763) published by Thalhammer (1900) probably refers to this species. Both species were often confused in the past (Haenni 1997). Our data represent the first reliable records of *S. cinctus* from Romania.

### Sylvicola (Sylvicola) fenestralis

(Scopoli, 1763)

#### Distribution

**Figure 4.**
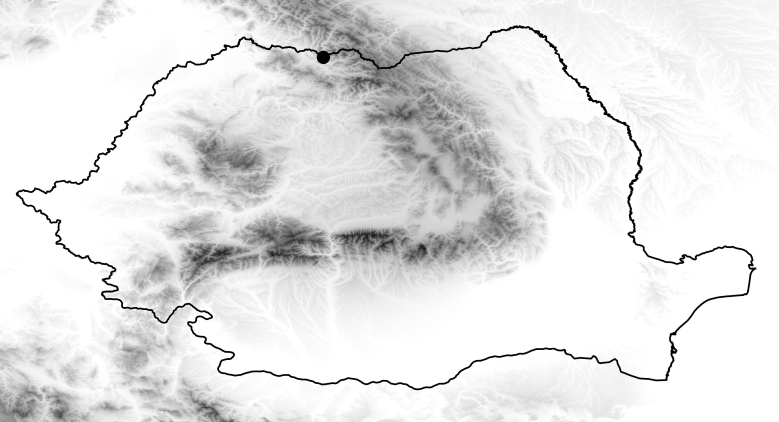
Collecting site of *Sylvicola fenestralis* in Romania based on literature (black dot).

Species distributed in almost all of European countries, but it is rarely collected. There is only a single literature record from the Maramures region (Fig. 4) (Pârvu 2003), which probably also refer to *S. cinctus*. This record waiting for confirmation.

## Identification Keys

### Key to Romanian *Sylvicola* Harris species

**Table:** 

1	Medial veins M_1_ and M_2_ arising from the discal cell at or almost at the same point and wing without dark spot at the apex (Fig. 5a).	subg. *Anisopus*, 2
–	Medial vein M2 arising from the discal cell separately between veins M_1_ and M_3_ and wing with dark spot at the apex (Fig. 5b​).	subg. *Sylvicola*, 3
2	Wing with dark band posterior of R_2+3_(Fig. 5a​). Hypoproct of male at apex shaped with two broad diverging lobes, gonocoxites at apex boadly rounded with a bulb on postero-lateral margin, parameres simply curved (Fig. 6a). Hypoproct of female almost triangular shaped with notch at its apex (Fig. 7a​).	S. (A.) punctatus (Fabricius, 1787)
–	Wing without dark marking posterior of R_2+3._ Hypoproct of male at apex shaped with two broad and short lobes, gonocoxites at apex narrowly rounded, parameres two times curved (Fig. 6b) and inside apex of gonocoxite with short and broad projection. Hypoproct of female short (Fig. 7b).	S. (A.) fuscatus (Fabricius, 1775)
3	Hypoproct broadly rounded, wider than high and with distal pubescent lobes; gonostyles with thickened base and without laterobasal projection; gonocoxites slightly diverging with angular projecting inwards (Fig. 8a). Hypoproct of female short, genital fork with two median, well sclerotized rods (Fig. 9a).	S. (S.) cinctus (Fabricius, 1787)
–	Hypoproct not such broadly rounded, equally wide as high, with posterior emargination and with median pubescent lobes; gonostyles narrower and with short laterobasal projection; gonocoxites clearly diverging with rounded projecting inwards(Fig. 8b). Hypoproct of female distinctly extended posteromedially, genital fork without median sclerotized rods (Fig. 9b​).	S. (S.) fenestralis (Scopoli, 1763)

## Discussion

In total 109 *Sylvicola* specimens were collected that belong to three species of which *Sylvicola cinctus* and *S. fuscatus* are new to the Romanian fauna. Four species of *Sylvicola* are now know from Romania. The occurrence of *Sylvicola zetterstedti* (Edwards, 1923) is to be excepted.

Romanian collection data, phenology and illustrations of male and female genitalia of the *Sylvicola* species are also available on the webpage of the Transylvanian Dipterological Working Group (www.transdiptera.com).

## Supplementary Material

XML Treatment for Sylvicola (Anisopus) punctatus

XML Treatment for Sylvicola (Anisopus) fuscatus

XML Treatment for Sylvicola (Sylvicola) cinctus

XML Treatment for Sylvicola (Sylvicola) fenestralis

## Figures and Tables

**Figure 5a.**
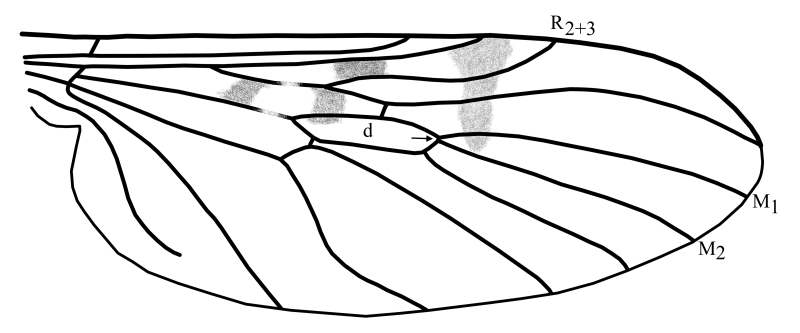
Sylvicola (Anisopus) punctatus (Fabricius, 1787)

**Figure 5b.**
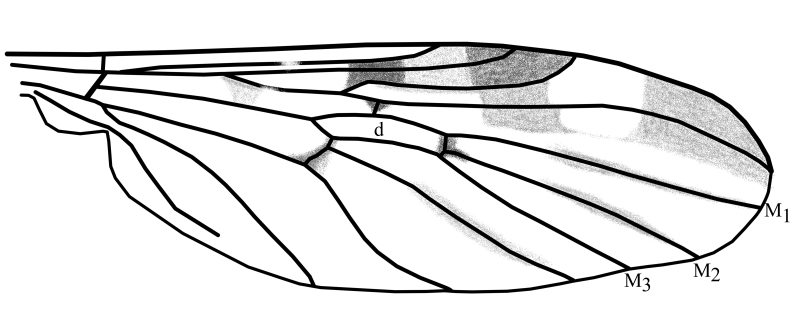
Sylvicola (Sylvicola) cinctus (Fabricius, 1787

**Figure 6a.**
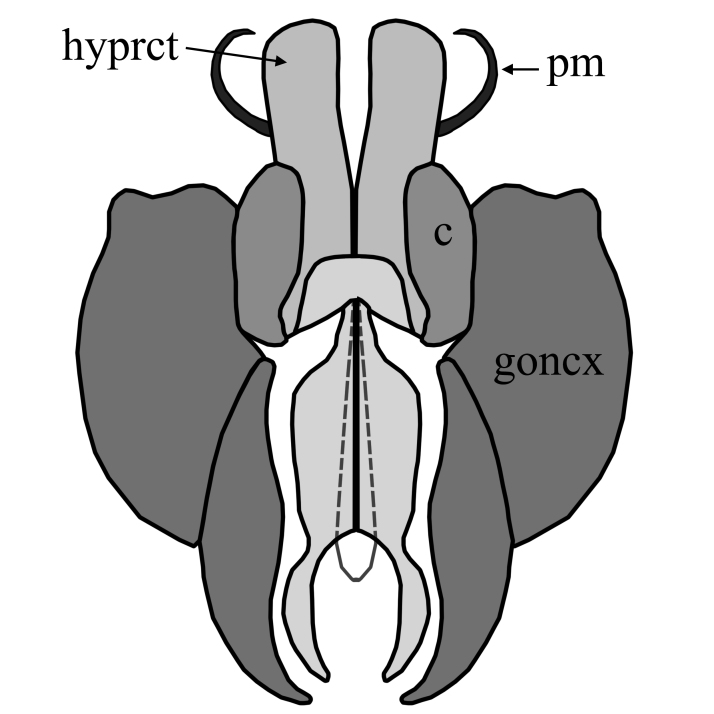
Sylvicola (Anisopus) punctatus (Fabricius, 1787)

**Figure 6b.**
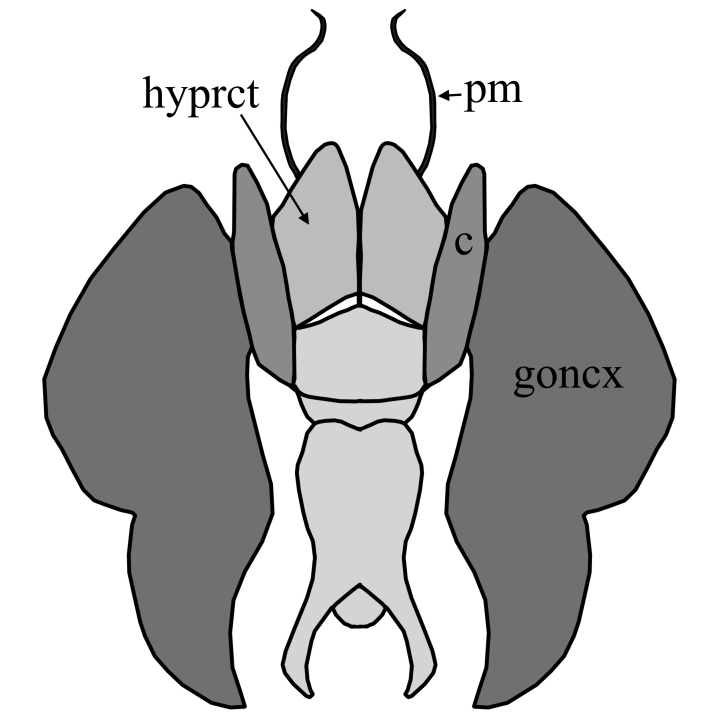
Sylvicola (Anisopus) fuscatus (Fabricius, 1775)

**Figure 7a.**
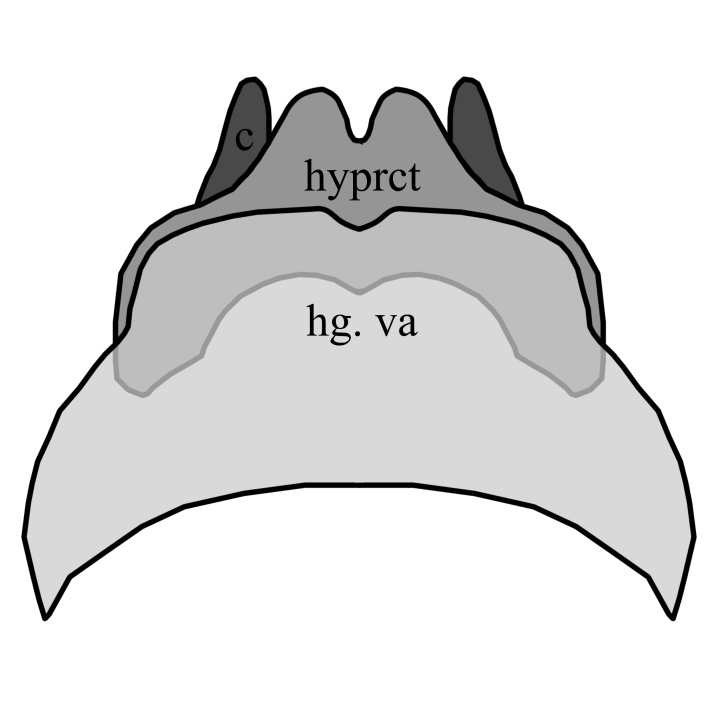
Sylvicola (Anisopus) punctatus (Fabricius, 1787)

**Figure 7b.**
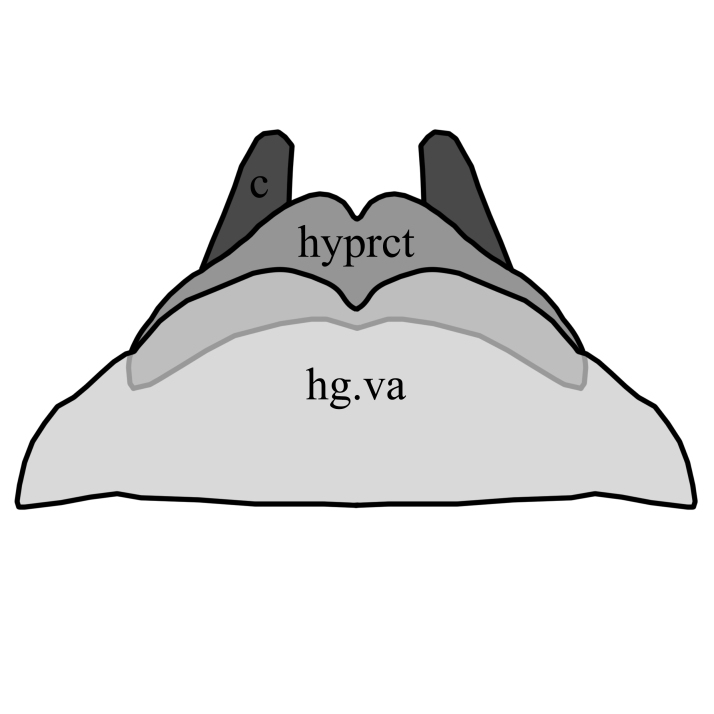
Sylvicola (Anisopus) fuscatus (Fabricius, 1775)

**Figure 8a.**
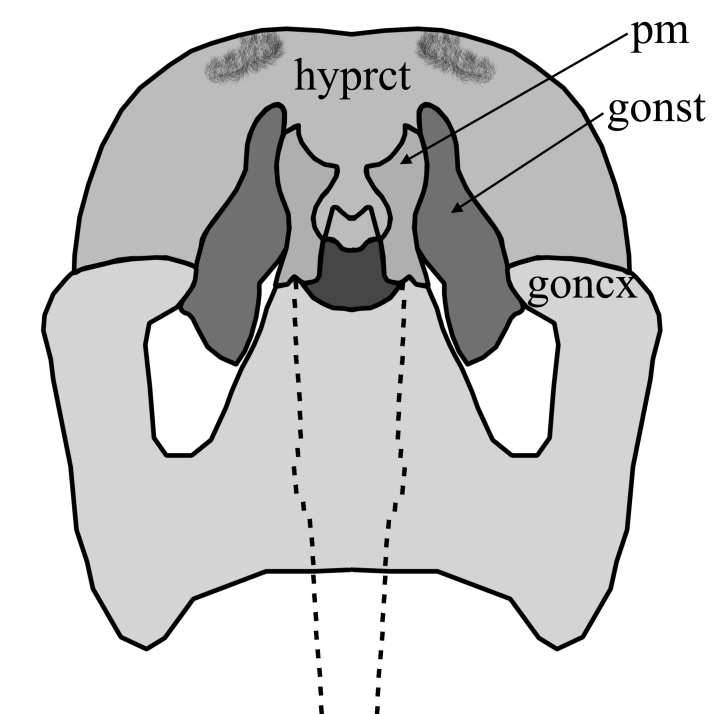
Sylvicola (Sylvicola) cinctus (Fabricius, 1787)

**Figure 8b.**
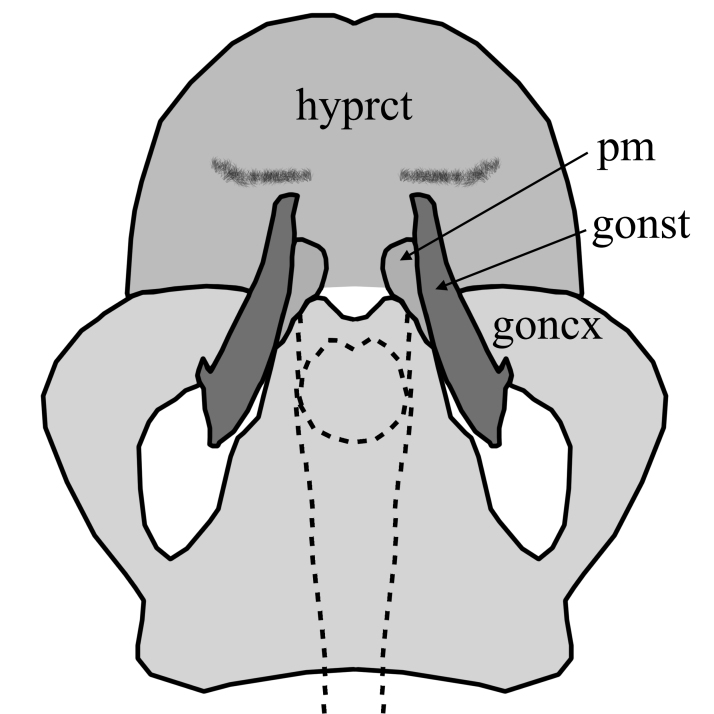
Sylvicola (Sylvicola) fenestralis (Scopoli, 1763)

**Figure 9a.**
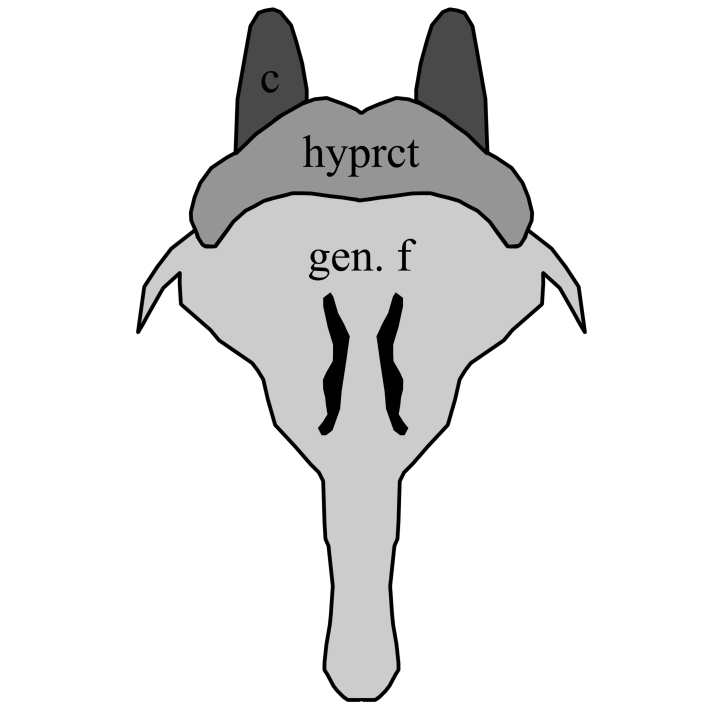
Sylvicola (Sylvicola) cinctus (Fabricius, 1787)

**Figure 9b.**
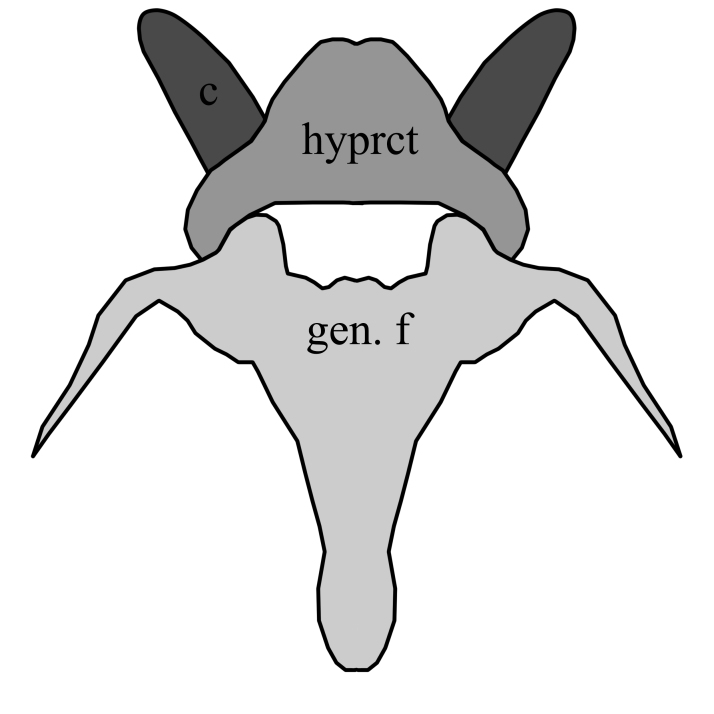
Sylvicola (Sylvicola) fenestralis (Scopoli, 1763)
